# The Fried Phenotype Is More Closely Associated With Dementia in Older Adults Than the FRAIL (Fatigue, Resistance, Ambulation, Illness, and Loss of Weight) Index

**DOI:** 10.7759/cureus.88094

**Published:** 2025-07-16

**Authors:** Fatih Atik, Suleyman Emre Kocyigit

**Affiliations:** 1 Internal Medicine, Faculty of Medicine, Balıkesir University, Balıkesir, TUR; 2 Geriatrics, Balıkesir University, Balıkesir, TUR

**Keywords:** ageing, dementia, frail frailty scale, frailty, fried phenotype, geriatric syndrome, prefrailty

## Abstract

Introduction: There is a close relationship between dementia and frailty in older adults. The aim of our study was to compare the relationship between the Fried and FRAIL (fatigue, resistance, ambulation, illness, and loss of weight) frailty scales and the frequency of dementia in elderly individuals.

Methods: A cross-sectional study was conducted on 558 patients who presented to the geriatric outpatient clinic between 2022 and 2024. The Fried and FRAIL frailty scales were used to assess frailty. On both scales, the groups were divided into three subgroups: frail, prefrail, and robust. The groups were also compared in terms of dementia frequency and other features. Correlations between frailty scores and the geriatric assessment test were examined. Logistic regression analysis was performed on the relationship between dementia frequency and frailty scores on both scales, independent of confounding factors.

Results: The mean age of 558 patients was 75.55 (6.83) years, and 69.7% were female. According to the FRAIL scale, the frailty rate was 24.7%, while according to the Fried scale, this rate was 36.1%. When the groups were compared on both scales, there were significant differences between age, gender, Parkinson's disease, Charlson Comorbidity Index, and geriatric syndromes. According to the FRAIL and Fried scales, the frequency of dementia was high in the frail group. Only in the Fried frailty index, the risk of dementia was significantly associated with frailty, independent of demographic characteristics, geriatric syndromes, and comorbidities.

Conclusion: In geriatric practice, frail patients diagnosed with the Fried frailty scale need to be carefully and comprehensively evaluated for cognitive impairment, but further studies are needed.

## Introduction

Frailty in older adults is a multifaceted geriatric syndrome characterized by decreased physiological reserves and increased vulnerability to stressors, leading to adverse health outcomes such as falls, disability, hospitalization, and mortality [1]. Frailty is also defined as a multisystem syndrome, although no single organ system is affected, nor is it isolated to a specific disease process [2]. Older people in acute care hospitals and nursing homes, those in low- or middle-income countries, and those who are socially vulnerable are all at greater risk of frailty [3]. Early identification and management of frailty are crucial. Frailty should be recognized as a distinct clinical syndrome in geriatric practice, and appropriate interventions should be implemented to reduce its progression and associated risks [4]. There is no consensus diagnostic method for frailty diagnosis; however, there are reliable, easily accessible, and appropriate methods for frailty screening in geriatric practice. Of these, the two most commonly used are the FRAIL (fatigue, resistance, ambulation, illness, and loss of weight) and Fried frailty scales. The FRAIL frailty scale includes fatigue, resistance, ambulation, illness, and loss of weight components, while the Fried frailty scale consists of unintentional weight loss, exhaustion, low physical activity, slow gait speed, and weak grip strength [5,6].

Dementia is a progressive neurodegenerative disorder that affects daily living activities [7]. The incidence of dementia increases with age, and it is closely associated with healthcare difficulties [8]. Therefore, early diagnosis of dementia is essential, and slowing down the progression of the disease with interventions such as lifestyle changes and drug therapy, adjustments in daily living activities, and effective comorbidity management are among the goals of geriatric practice [9]. Dementia itself is a geriatric syndrome and is associated with many other geriatric syndromes, including sarcopenia, malnutrition, and polypharmacy. In addition, the number of geriatric syndromes is higher in patients with dementia [10]. It is well known that physical frailty and dementia are closely related. Physical frailty may be an important early warning sign of dementia and a target for interventions [11]. Frailty may represent a useful upstream target for behavioral and social approaches to dementia prevention [12].

There are not enough studies to determine which frailty scale is most associated with dementia in the literature. The aim of our study was to examine and compare the relationships between two frailty indices, the FRAIL and Fried scales, and dementia.

## Materials and methods

Study design

This study included 558 older adults who applied to the Balikesir University Hospital outpatient geriatric clinic between December 2022 and December 2024. It was designed as a cross-sectional and observational study. After informed written consent was obtained from all patients, a comprehensive geriatric assessment (CGA), including frailty assessment, was performed.

Inclusion criteria

The study included individuals over 65 years of age who did not meet any of the exclusion criteria.

Exclusion criteria according to the Fried and FRAIL frailty assessment tools

To ensure the reliability and validity of frailty assessments, specific exclusion criteria were defined separately for the Fried frailty phenotype and the FRAIL index, based on their methodological differences and clinical applicability.

Exclusion Criteria for the Fried Frailty Phenotype

This tool includes physical performance measures such as grip strength and gait speed; therefore, the following conditions that significantly impair physical function were excluded: severe or symptomatic anemia (hemoglobin <10 g/dL); New York Heart Association (NYHA) class III or IV heart failure; critical cardiac valve stenosis; acute or chronic renal insufficiency; decompensated hepatic failure; severe carotid artery stenosis and/or coronary artery stenosis; recent (within the past month) cerebrovascular accident, myocardial infarction, or lower extremity fracture; hypotensive shock; bradycardia or tachycardia at the time of examination; severe dehydration; significant electrolyte imbalance; acute hemorrhage; severe metabolic acidosis; sepsis; active malignancy; immobility due to severe osteoarthritis; inability to walk due to neuromuscular diseases; and acute mental status changes (e.g., delirium).

Exclusion Criteria for the FRAIL Index

Since the FRAIL Index relies on self-reported functional status and comorbidities, conditions affecting cognition, communication, or the ability to provide reliable information were prioritized for exclusion. These include the following: acute mental status changes (e.g., delirium); disturbed consciousness or impaired attention; hemodynamic instability (e.g., shock, severe hypotension, marked bradycardia, or tachycardia); terminal illnesses with rapid clinical deterioration (e.g., sepsis, advanced malignancy); severe dehydration or metabolic derangements affecting sensorium; sensory impairments (e.g., profound hearing or visual loss) interfering with questionnaire completion; neuromuscular diseases or immobility are likely to confound self-reported fatigue or physical function; and recent major trauma (e.g., lower extremity fracture) within the past month.

Patient characteristics and CGA parameters

Demographic features, including age, gender (female), education level, and body mass index (kg/m^2^) values, and geriatric syndromes, including polypharmacy (> five medication use), probable sarcopenia, geriatric depression, recurrent falls in the last year, and malnutrition, were recorded. Comorbid diseases, including hypertension, diabetes mellitus, ischemic heart disease, congestive heart failure of NYHA grade 1 or 2, chronic obstructive pulmonary disease, osteoporosis, and Parkinson’s disease, were screened. The Charlson Comorbidity Index was calculated for all patients. Patients underwent CGA, including the Mini-Mental State Examination (MMSE), Tinetti Performance Oriented Mobility Assessment (POMA), timed up-and-go test, Barthel (basic) activities of daily living (BADL) index, and Lawton-Brody instrumental activities of daily living (IADL). Dementia or major cognitive impairment was diagnosed according to the Diagnostic and Statistical Manual of Mental Disorders, Fifth Edition (DSM-5) criteria.

Frailty assessment

Two frailty scales were applied to all participants in the CGA perspective. The first of these is the FRAIL frailty scale, which includes the following questions: (1) Are you fatigued? (2) Are you unable to walk up one flight of stairs? (3) Are you unable to walk one block? (4) Do you have more than five illnesses? (5) Have you lost more than 5% of your weight in the last six months? If three or more of these components were present, the subject was defined as frail; if one or two components were present, they were defined as prefrail; if none were present, they were defined as robust [5].

The other frailty scale measured was Fried’s frailty scale. The components of this scale are weakness, slowness, low level of physical activity, exhaustion, and weight loss. Patients were divided into three groups according to their frailty scales: frail (three to five points), prefrail (one to two points), or robust (zero points) [6].

Statistical analysis

Categorical variables are shown as percentages (%). For continuous variables, a normality test was performed using the Kolmogorov-Smirnov test. Since not all variables were normally distributed, relevant variables were expressed as median (interquartile range). The chi-square test was used to compare categorical variables. Mann-Whitney U (between binary groups) or Kruskal-Wallis (between multiple groups) tests were used to compare continuous variables, depending on the number of groups. The Spearman correlation test was applied for the correlation of Fried and FRAIL frailty scores for continuous variables. Multivariable logistic regression analysis was performed on the relationship between the FRAIL and Fried subgroups and dementia. Variables that showed a significance level of p < 0.10 in univariate analysis, or were considered clinically relevant, were included in the multivariate models. Odds ratios (ORs) were calculated with 95% confidence intervals (CIs). For odds ratios, unadjusted ORs were calculated in model 0. The OR was adjusted for demographic characteristics in model 1. Model 2 was generated and adjusted for model 1, plus the Charlson Comorbidity Index, osteoporosis, and Parkinson’s disease. Model 3 was based on model 2 and included additional adjustments for geriatric syndromes. In statistical analyses, a p-value of less than 0.05 indicated significance. Statistical analyses were performed using SPSS version 22.0 package program (IBM Corp., Armonk, NY).

## Results

When 558 participants were evaluated, their mean age was 75.55 (6.83) years, and 69.7% of all patients were female. According to the FRAIL frailty scale, the frequency of frail, prefrail, and robust individuals was 24.7%, 55.6%, and 19.7%, respectively. Similarly, based on the Fried frailty scale, the frequency of frail, prefrail, and robust older adults was 36.1%, 43.7%, and 20.2%, respectively.

When the groups were compared as frail, prefrail, and robust according to both frailty scales, age and years of education were statistically different between groups. The frequency of female gender and the rate of geriatric syndromes differed between groups according to both frailty scales (p < 0.05; Table 1). The frequency of geriatric syndromes was higher in frail individuals according to both the FRAIL and Fried frailty scales (p < 0.05). When comorbidities were compared, the Charlson comorbidity score and Parkinson's disease frequency were statistically significant among the three groups on both frailty scales (p < 0.05). Only the frequency of osteoporosis was statistically significant among these three groups according to the Fried frailty scale (p < 0.05) (Table 1).

**Table 1:** Comparison of demographic characteristics, comorbidities, and geriatric syndrome frequencies of frailty groups according to the FRAIL and Fried frailty scales. FRAIL: fatigue, resistance, ambulation, illness, and loss of weight; BMI: body mass index; CCI: Charlson Comorbidity Index; COPD: chronic obstructive pulmonary disease; IQR: interquartile range. * The chi-square test was performed to compare categorical data between groups. The test value used for categorical data in the table is the chi-square value. The Kruskal-Wallis test was used for continuous data, including age, years of education, body mass index, and Charlson Comorbidity Index. The test value used for age, years of education, body mass index, and Charlson Comorbidity Index is the Kruskal-Wallis test value.

	FRAIL score					Fried score				
	Frail (n = 130)	Prefrail (n = 310)	Robust (n = 118)	*Test value	p-value	Frail (n = 201)	Prefrail (n = 245)	Robust (n = 112)	*Test value	p-value
Demographic features										
Age, median (IQR)	79 (9)	75 (11)	73 (9)	30.24	<0.001	79 (9)	75 (11)	72 (7)	58.09	<0.001
Gender (female, %)	81.2	70.0	54.5	20.56	<0.001	21.0	32.2	42.9	16.97	<0.001
Education year, median (IQR)	5 (5)	5 (0)	5 (6)	39.04	<0.001	5 (3)	5 (0)	5 (3.75)	32.02	<0.001
BMI (kg/m^2^), median (IQR)	27.1 (8.1)	26.3 (7.7)	26.2 (5.4)	0.20	0.902	27.05 (7.7)	26.2 (6.9)	26.8 (7.6)	0.98	0.612
Comorbidities (%)										
Hypertension	74.6	73.9	61.8	6.59	0.037	72.0	71.5	71.4	0.02	0.991
Diabetes mellitus	40.6	44.2	35.5	2.62	0.269	40.0	46.3	35.7	3.96	0.138
Coronary artery disease	18.8	20.6	20.0	0.19	0.907	21.0	17.8	24.1	2.02	0.363
Congestive heart failure	15.2	9.7	8.2	3.99	0.135	13.5	9.5	8.9	2.33	0.311
COPD	8.0	4.2	4.5	2.88	0.236	6.0	5.4	2.7	1.74	0.419
Osteoporosis	35.3	25.4	17.9	5.36	0.068	36.8	24.6	9.7	15.20	<0.001
Parkinson’s disease	13.8	6.1	1.8	14.26	<0.001	16.0	2.9	0.9	36.47	<0.001
CCI, median (IQR)	1 (2.25)	1 (2)	1 (2)	9.17	0.010	1 (2.75)	1 (2)	1 (2)	9.44	0.009
Geriatric syndromes (%)										
Probable sarcopenia	69.1	36.8	24.5	58.07	<0.001	75.4	32.2	5.4	161.86	<0.001
Recurrent falls	62.3	40.3	32.7	26.12	<0.001	60.0	34.3	36.6	32.50	<0.001
Malnutrition	67.9	24.5	9.1	115.31	<0.001	60.5	19.8	6.3	125.71	<0.001
Polypharmacy	71.7	67.7	56.4	6.98	0.030	75.0	64.0	57.1	11.55	0.003
Geriatric depression	69.6	44.5	20.0	61.08	<0.001	68.0	38.8	21.4	71.21	<0.001

When evaluated according to the FRAIL frailty scale, the frequency of dementia was higher in frail individuals than in the robust and prefrail groups (p < 0.05). In parallel, the MMSE test scores were lower in the frail group compared to the other two groups (p < 0.05). The frequency of dementia and the MMSE test scores were statistically similar between the prefrail and robust groups (p > 0.05). Timed up and go (TUG) duration was the longest in the frail group, while BADLs and IADLS were the lowest in the frail group (p < 0.05). These three parameters were statistically different when the frail, prefrail, and robust groups were compared in pairs (p < 0.05). According to the Fried frailty scale, the frequency of dementia was higher in frail individuals than in the robust and prefrail ones (p < 0.05). The rate of dementia was determined to be higher in the prefrail group than in the robust group (p < 0.05). While the MMSE test score was lower in the frail group than in the other two groups, the MMSE test score was also lower in the prefrail group than in the robust group (p < 0.05). TUG duration was the longest in the frail group and the shortest in the robust group; BADLs and IADLS were the lowest in the frail group and the highest in the robust group (p < 0.05) (Table 2).

**Table 2:** Comparison of the frequency of dementia, neurocognitive, mobilization, and ADLs tests at the frail, prefrail, and robust groups according to the FRAIL and Fried frailty scales. ADLs: activities of daily living; FRAIL: fatigue, resistance, ambulation, illness, and loss of weight; BADLs: basic activities of daily living; IADLs: instrumental activities of daily living; IQR: interquartile range; MMSE: Mini-Mental State Examination; TUG: timed up and go. p1: Comparison between the frail and robust groups. p2: Comparison between the prefrail and robust groups. p3: Comparison between the frail and prefrail groups. * The chi-square test was applied to compare the frequency of dementia between the two groups. The test value used for categorical data in the table is the chi-square value. The Mann-Whitney U test was used for continuous data, including MMSE, TUG duration, basic and instrumental ADLs, because it was encountered between two groups and the data did not conform to normal distribution. The test value used for MMSE, TUG duration, and basic and instrumental ADLs is the Z value.

FRAIL score						
	Frail group	Prefrail group	Robust group	p_1,_ *test score	p_2_, * test score	p_3, _*test score
Dementia (%)	36.2	21.4	19.1	0.003, 8.80	0.614, 0.25	0.001, 10.98
MMSE, median (IQR)	21 (10)	25 (8)	25 (8)	<0.001, -3.61	0.245, -1.16	0.001, -3.27
TUG duration (sec), median (IQR)	22.4 (24.02)	15.4 (7.56)	13.37 (4.42)	<0.001, -10.21	<0.001, -4.77	<0.001, -9.68
BADLs, median (IQR)	75 (26.25)	90 (10)	100 (5)	<0.001, -12.64	<0.001, -9.58	<0.001, -10.91
IADLs, median (IQR)	12 (11)	20 (6)	21 (4.25)	<0.001, -9.13	<0.001, -3.33	<0.001, -9.07
Fried score						
Dementia (%)	45.7	14.5	7.1	<0.001, 49.17	0.049, 3.88	<0.001, 52.30
MMSE, median (IQR)	19 (11)	25 (6)	27 (5)	<0.001, -7.72	0.007, -2.71	<0.001, -7.42
TUG duration (sec), median (IQR)	23.38 (18.06)	14.3 (5.7)	12.61 (3.97)	<0.001, -12.41	<0.001, -6.14	<0.001, -11.81
BADLs, median (IQR)	80 (25)	95 (5)	97.5 (5)	<0.001, -12.14	<0.001, -5.52	<0.001, -11.81
IADLs, median (IQR)	12 (10)	20 (4)	22 (3)	<0.001, -12.85	<0.001, -5.87	<0.001, -12.66

In Spearman correlation analysis, there was a positive correlation with TUG duration and CCI in both frailty scores and a negative correlation with ADLs and MMSE scores in both FRAIL and Fried frailty scores (p < 0.05). While the correlation value of the FRAIL frailty test score with MMSE was r = -0.184 (p < 0.001), the correlation value of the Fried frailty scale with MMSE was observed as r = -0.442 (p < 0.001) (Table 3).

**Table 3:** Correlation of the Fried and FRAIL frailty test scores with MMSE, CCI, ADLs, and balance-walking test values. CCI: Charlson Comorbidity Index; ADLs: activities of daily living; FRAIL: fatigue, resistance, ambulation, illness, and loss of weight; BADLs: basic activities of daily living; IADLs: instrumental activities of daily living; MMSE: Mini-Mental State Examination; TUG: timed up and go. * For the correlation of continuous data with frailty scores, the Spearman correlation test was applied since the data did not conform to a normal distribution. The *r* correlation coefficient was calculated.

FRAIL group		
	r correlation	p-value
MMSE	-0.184	<0.001
BADLs	-0.643	<0.001
IADLs	-0.428	<0.001
TUG duration	0.489	<0.001
CCI	0.128	0.002
Fried group		
MMSE	-0.442	<0.001
BADLs	-0.614	<0.001
IADLs	-0.653	<0.001
TUG duration	0.630	<0.001
CCI	0.131	0.002

In the multivariable logistic regression analysis, when the robust group was taken as the reference category according to the FRAIL scale, a relationship was found with the frequency of dementia in the frail group in model 0 and model 1 (p < 0.05). In models 2 and 3, the frail group was not associated with dementia (p > 0.05). In the Fried frailty scale, prefrailty and frailty were associated with dementia in the unadjusted model (OR: 2.19, 95% CI: 1.02-4.90; p = 0.048 and OR: 10.95, 95% CI: 5.06-23.68; p < 0.001, respectively). In adjusted model 1, the prefrail group and frail group were related to dementia (OR: 2.48, 95% CI: 1.09-5.64; p = 0.030 and OR: 13.79, 95% CI: 5.98-31.82; p < 0.001, respectively). In models 2 and 3, only the frail group was associated with dementia (OR: 6.09, 95% CI: 2.10-17.68; p = 0.001 in model 2 and OR: 5.93, 95% CI: 1.55-22.64; p = 0.009) (Table 4).

**Table 4:** Investigation of the relationship between dementia frequency and both frailty scales, independent of confounding factors, using multivariable regression analysis. FRAIL: fatigue, resistance, ambulation, illness, and loss of weight. To examine the interaction of frailty status with the presence of dementia in both the Fried frailty scale and the FRAIL frailty scale, multivariable logistic regression analysis was used, taking the robust group as the reference category. Model 0: Unadjusted. Model 1: Adjusted for age, gender, and education year. Model 2: Adjusted for model 1 plus Charlson Comorbidity Index, osteoporosis, and Parkinson’s disease. Model 3: Adjusted for model 2 plus geriatric syndromes, including probable sarcopenia, malnutrition, recurrent falls, polypharmacy (>five medication use), and geriatric depression.

FRAIL score					
		Odds ratio	95% CI	p-value	R^2^
Model 0	Prefrail group	1.15	0.66-1.99	0.615	0.02
	Frail group	2.40	1.33-4.33	0.003	
Model 1	Prefrail group	1.19	0.67-2.11	0.537	0.15
	Frail group	2.24	1.18-4.24	0.013	
Model 2	Prefrail group	0.96	0.45-2.06	0.934	0.15
	Frail group	1.11	0.44-2.76	0.817	
Model 3	Prefrail group	0.74	0.32-1.68	0.481	0.35
	Frail group	0.44	0.15-1.31	0.143	
Fried score					
Model 0	Prefrail group	2.19	1.02-4.90	0.048	0.15
	Frail group	10.95	5.06-23.68	<0.001	
Model 1	Prefrail group	2.48	1.09-5.64	0.030	0.29
	Frail group	13.79	5.98-31.82	<0.001	
Model 2	Prefrail group	1.39	0.50-3.85	0.527	0.33
	Frail group	6.09	2.10-17.68	0.001	
Model 3	Prefrail group	1.58	0.51-4.92	0.424	0.60
	Frail group	5.93	1.55-22.64	0.009	

## Discussion

In this study, examining the relationship between the two most commonly used frailty screening methods in the literature, i.e., FRAIL and Fried scales, and the frequency of dementia, the frequency of dementia was higher in frail individuals than in prefrail and robust groups on both frailty scales. However, when adjusted for confounding factors, the frequency of dementia was higher in frail older adults in the group where only the Fried frailty scale was used. In addition, a higher correlation was observed with the MMSE test and the Fried score than with the FRAIL score.

Frailty is a geriatric syndrome that increases in frequency in older adults and is associated with adverse outcomes in the elderly. Frailty assessment is an integral part of a comprehensive geriatric assessment. Frailty prevalence varies in the literature in older adults. Frailty prevalence increases with age, regardless of the screening method [13]. When the Fried frailty scale is used, the prevalence of frailty can vary between 4% and 59% in community-dwelling older adults [13]. In a systematic review conducted in the Chinese older population, the prevalence of frail and prefrail older adults was found to be around 10.1% and 43.9%, respectively [14]. In our study, the prevalence of frailty was found to be 24.7% according to the FRAIL frailty index and 36.1% according to the Fried index. The prefrailty rate was 43.7% according to the Fried index and 55.6% on the FRAIL scale. Although our findings are parallel to the literature, both frailty and prefrailty frequencies are quite variable in the literature. The reasons for these may be the patient population (community-based, inpatient, or nursing home residents), the influence of race or the country of study, and differences in applied frailty scales. The relatively low proportion of robust individuals (around 20%) in our study can be explained by several factors. First, the study population consisted of older adults who were referred to a geriatric outpatient clinic, suggesting that they may already have had health concerns or functional limitations. This clinical population may not reflect the general community-dwelling older adult population, in which robustness is typically higher. Additionally, high rates of comorbidities, polypharmacy, and potential functional decline commonly observed in such settings could have contributed to the lower robustness rate.

Frailty, a dynamic and reversible process in older adults, is also associated with many other age-related disorders or geriatric syndromes. Frailty plays a leading role in the management and treatment choices of many diseases in older adults. Although frailty is more common in community-dwelling older adults due to age, it can also be present in some specific disease processes. For example, it has been shown in the literature that frailty may have a bidirectional relationship with geriatric depression in older adults [15]. A close relationship exists between multimorbidity and frailty [16]. The literature emphasizes the relationship between frailty and cardiovascular disease processes. There may be an 83% frequency of frailty of varying degrees in older adults with heart failure, and it is an important predictor of mortality in this population [17]. Moreover, it is emphasized that the risk of frailty is significantly increased in individuals diagnosed with stroke and that it has prognostic importance [18]. In our study, although no significant difference was detected in terms of frailty among comorbidities except for Parkinson's disease, we have shown that the Charlson Comorbidity Index is higher in frail older people and that the burden of comorbidity might be related to frailty. This emphasizes that the burden of comorbidity may be more important than a single chronic disease in terms of frailty in community-dwelling older adults.

Dementia is a significant cause of mortality and morbidity, and Alzheimer's disease, the most common type of dementia, is closely associated with frailty as well as falls, polypharmacy, and delirium in older adults [19]. Frailty management is an important part of geriatric practice, especially in patients diagnosed with dementia. Cognitive frailty is most commonly defined as the simultaneous presence of physical frailty and mild cognitive impairment, excluding dementia, based on the International Academy on Nutrition and Aging (IANA)/International Association of Gerontology and Geriatrics (IAGG) consensus from 2013 [20]. The mechanisms potentially linking physical and cognitive impairment involve vascular dysfunction, chronic inflammation, and shared brain pathologies such as white matter hyperintensities and subcortical atrophy [21]. The risk of dementia during follow-up is approximately 3.6 times higher in the high-frailty group, regardless of genetic risk, according to the 49-item frailty index [22]. Inflammation is a key biological mechanism linking physical frailty (as assessed by the Fried scale) to dementia, reflecting systemic and neuroinflammatory processes involved in both conditions [23]. Social isolation is a prominent social mechanism that may explain the stronger association between the Fried frailty phenotype and dementia, as reduced social engagement is both a risk factor for cognitive decline and a contributor to physical frailty [24]. Therefore, frailty screening is essential in individuals diagnosed with dementia, and it is necessary to choose a frailty screening method with care. In our study, we showed that frailty on the Fried frailty scale can be more clearly associated with dementia risk, independent of confounding factors. We also emphasized that the MMSE score and the evaluation scores on the Fried frailty scale are more negatively correlated than assessment scores on the FRAIL scale. In this respect, the Fried frailty index seems to be more reliable in frailty assessment in elderly patients diagnosed with dementia than the FRAIL scale.

There are several studies in the literature comparing the Fried frailty scale with the FRAIL index. When these studies are examined in general, it is emphasized that the FRAIL frailty index is easy to use, less expensive, and more reliable, and this comparison is usually made by taking the Fried frailty index as a reference. This comparison can be made in the general population as well as in special patient groups. For instance, in a study conducted on 293 cancer survivors, it is emphasized that the FRAIL index is comparable to the Fried index and is an index that can be recommended for frailty in this group [25]. In addition, it has been shown that the Fried frailty index has significant differences between the frail, prefrail, and robust stages in the social, psychological, and physical function domains [26]. Based on the Fried frailty index, frailty is associated with geriatric syndromes such as orthostatic hypotension as well as dementia [27]. There is no sufficient study in the literature in which the frailty index is superior in patients with dementia. In our study, it was highlighted that the Fried frailty index might be more valuable than the FRAIL index in the frailty assessment of individuals with dementia, independent of confounding factors. There may be many reasons for this situation. One of these is that the Fried frailty index has more objective components; in particular, it includes the components of slowing down walking speed and decreased muscle grip strength, which are conditions that increase the risk of dementia. It is known that sarcopenia is associated with probable Alzheimer's disease and probable dementia with Lewy bodies [28]. It is well known that this cognitive and motor decline has common pathophysiology, such as mitochondrial dysfunction, cellular senescence, genomic instability, epigenetic changes, and inflammation, and that most of these mechanisms are involved in the etiopathogenesis of frailty [29]. These findings reveal the importance of objectively assessing low walking speed in frailty assessment. The decrease in muscle strength also increases the risk of dementia, and especially the decrease in upper extremity muscle strength measured in the Fried frailty index may cause executive function deterioration as a result of the decrease in reciprocal connectivity in the frontoparietal network [30]. In addition, according to self-reported findings, the reliability of the FRAIL index, which is a screening criterion, in the elderly with cognitive dysfunction is also questionable. There was an association between the FRAIL index and dementia in models 0 and 1, probably due to this reason.

The strength of our study is that it was the first study to compare frailty scales, including the Fried phenotype and the FRAIL index, in patients with dementia and to demonstrate this relationship independently of confounding factors.

There are also several limitations to this study. First, it is not easy to establish a causal relationship because it is a cross-sectional study. Second, comparisons with dementia subtypes could not be made. Third, multidimensional or other frailty indices could not be used in the evaluation.

## Conclusions

Frailty assessment in older adults diagnosed with dementia in geriatric practice is quite important in terms of dementia management. Frailty screening methods that include more objective measurements seem to be more valuable in older adults with cognitive dysfunction. The Fried frailty index appears to be more associated with the prevalence of dementia than the FRAIL scale. However, longitudinal, randomized controlled studies with larger sample sizes are needed to confirm this relationship.
